# Design of a multifunction novel flexible fault current limiter for AC distribution network

**DOI:** 10.1371/journal.pone.0245956

**Published:** 2021-04-08

**Authors:** Yao Liu, Lin Guan, Zhe Tan, Kun Yang, Fang Guo, Yong Chen, Renliang Liu, Feng Zheng

**Affiliations:** 1 School of Electric Power, South China University of Technology, Guangzhou, Guang Dong, China; 2 Zhuhai Power Supply Company, State Grid Corporation of China, Zhuhai, Guang Dong, China; 3 College of Automation, Foshan University, Foshan, Guang Dong, China; 4 School of Electrical Engineering and Automation, Fuzhou University, Fuzhou, Fujian, China; University of Science and Technology Beijing, CHINA

## Abstract

Based on the separation voltage type of cascaded H bridge-modular multilevel converters (CHB-MMC) and current predictive model control (CPMC) technology, a novel flexible fault-current limiter (NFFCL) is firstly proposed for restraining the negative impact of the distribution network’s disturbance in this paper. When a disturbance occurs, the inner-loop CPMC of the multilevel converters establish the value function to offer the specific current, thus increasing the voltage deviation at both ends of the series capacitor or generating reverse harmonic compensation voltage. In that case, three single-phase MNFFCLs can be regarded as variable voltage sources to eliminate the negative effects of faults or harmonics. Owing to the multi-capacitance series structure, the maximum voltage drops of the single capacitance can be predetermined by the number of capacitors. And with the low voltage drop of single capacitance, the output current of the CHB-MMC can also be controlled within an acceptable range. Through the simulation results, the disturbance’s negative impact on the non-fault area can be eliminated almost 100%.

## Introduction

Due to the continuous expansion of the distribution network, the demand for safe and stable operation is increasing, especially in the case of failure condition [1]. And with the access of a large number of distributed power sources, the fault current of the distribution network increases sharply, even exceeding the breaking capacity of the circuit breaker. At present, for reducing the negative effects from fault current on the non-fault area in distribution network, the fault current limiter (FCL) is widely adopted [2, 3]. According to current limiting targets, FCL can be divided into two categories. One is used to inhibit the short-circuit current and ensure the safety and reliability of electrical equipment. The other is to cut off the short-circuit current directly, which is similar to the circuit breaker. Among them, the former has received extensive attention in research, especially superconducting and power electronic FCL [4, 5]. However, when a failure occurs, the superconducting FCL (SFCL) takes a long time to recover and needs to dissipate heat. Therefore, these shortcomings hinder the development of SFCL. Compared with SFCL, the power electronic FCLs (such as variable impedance type of FCL, resonance type of FCL and bridge type of FCL) have become the focus of current research, because of their flexible control, good action repeatability, fast breaking speed, small size and so on [6–9].

However, the lack of voltage and current withstand level of a single power electronic equipment limits the development of power electronic FCLs, which also brings great challenges to the research on the limitation of short-circuit fault current in medium and high voltage distribution networks [10]. In order to resolve these problems and enhance the transient performance of distribution network, the multi-level and multi-inverter techniques have been adopted [11–14]. In [11, 12], the multiphase LLC converter was proposed to achieve a great current-sharing performance without additional components or control, so that the overcurrent of single power electronic devices can be suppressed. For solving the problem of the overvoltage, the modular multilevel converter (MMC) was adopted in [13, 14]. Through the series sub-module (SM) capacitor, the average capacitor voltage of MMC is significantly reduced. And in [15], MMC was used to enhance the low voltage ride through (LVRT) capacity of wind energy conversion system. For eliminating the influences of the current injection amplitude on sub module voltages and arm currents, a trade-off method was given to optimize system design for MMC control system. In [16], two new SM circuit configurations as well as a hybrid design methodology to embed the dc-fault-handling capability in the MMC for high-voltage direct current systems were proposed. By combining the features of various SM configurations, the dc-fault current paths through the freewheeling diodes are eliminated and the dc-fault current is enforced to zero. However, no matter the methods in [15] or in [16], they were mainly related to the fault of the dc system. In [17], for improving the transient performance of AC distribution network in the fault conditions, three single-phase cascaded H-bridge multi-level converters and the voltage feedback control were adopted to establish a flexible current limiting method. Whereas, owing to the insufficient withstand voltage of single capacitor, this method was still not suitable for medium and high voltage distribution networks. And if they only work under malfunctioning conditions, the device utilization will be greatly reduced.

In view of the deficiency of the above research, a multifunction novel flexible fault current limiter (MNFFCL) is proposed to enhance the dynamic performance of AC multi-source distribution network in this paper. Three single-phase MNFFCLs are installed on both sides of transmission line. When a fault occurs in a multi-source distribution network, the voltage drop of a single capacitor can be kept at the preset value by establishing the value function of the CPMC controller of the multilevel converters. And because a plurality of capacitors is connected in series to the transmission line, the overvoltage of the H-bridge converter and the overcurrent flowing through the HBMMC can be eliminated. Under normal operation, it is the goal of MNFFCLs to eliminate the harmonic voltage in the transmission line. Therefore, according to the standard voltage and the actual voltage of the power grid, the value function of CPMC can be established.

Section II presents the topological circuit’s structure and control strategy of HB-MMC system. Section III describes the transient analysis of multi-source distribution network with HB-MMC. Section IV constructs ac multi-source distribution network model with NFFCLs in MATLAB. Section V makes a summary for the proposed method and draws relevant conclusions.

## HB-MMC system modeling

**Fig 1:**
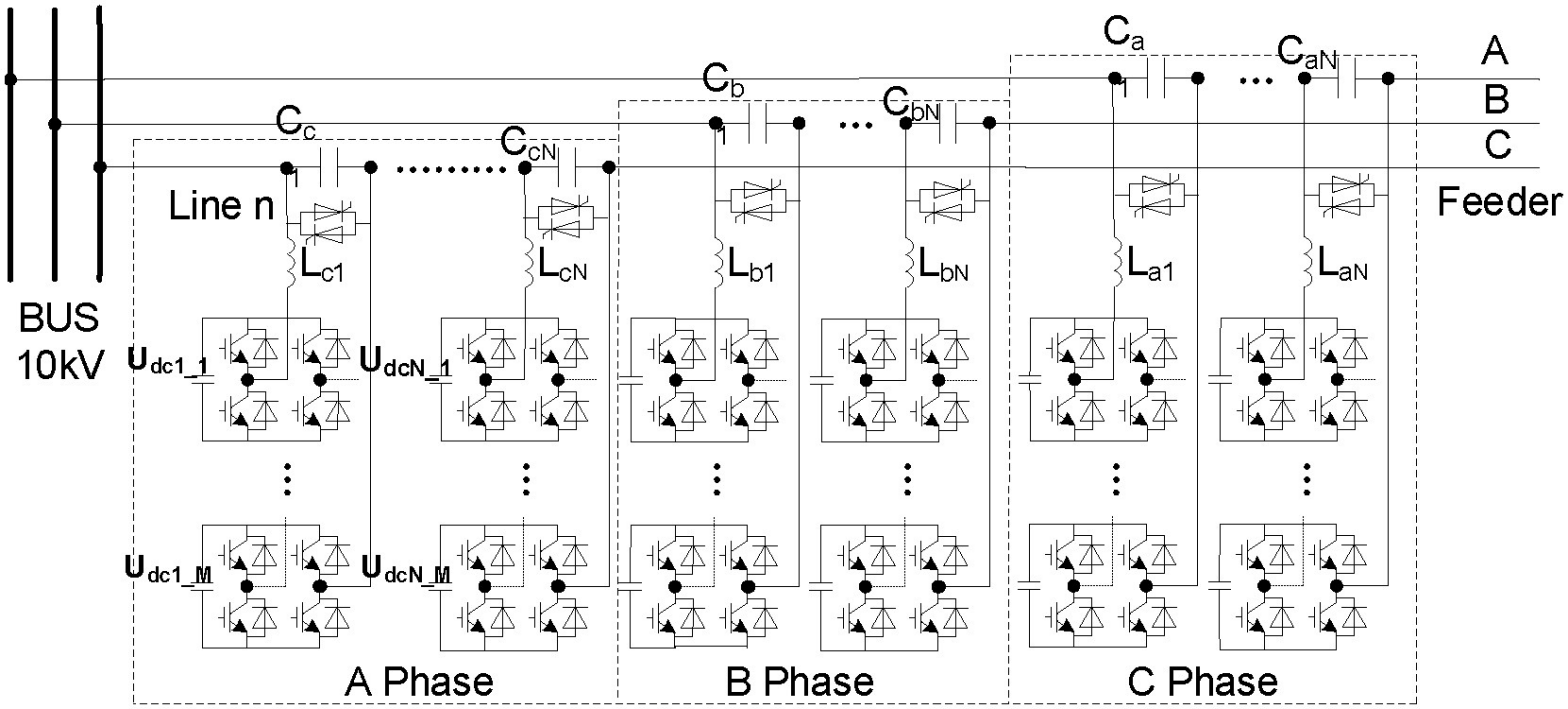
Schematic diagram of NFFCL.

Fig 1 shows the topological circuits’ structure of NFFCL which includes cascade HB-MMC, capacitances and inductance. Here, C_a/b/c_ and L_a/b/c_ are the three-phase capacitances and inductances, respectively. N and M respectively represent the number of series capacitances and the number of cascade H-bridge converter. U_dc_ is DC voltage of H bridge converter. The main goal of the cascade H bridge converters is to provide the current corresponding to the preset voltage drop of a single capacitor through four switches. When fault happens, the voltage of non-fault area can be quickly improved to the pre-set value. In order to reduce the harmonic from the cascade H bridge converters, the additional inductance, *L*, is in series with them, so that the superior fundamental voltage of series capacitance can be obtained. It can be seen from Fig 1 that the cascade H-bridge converter can be significantly enhanced because the number of series capacitors can be easily increased so as to improve the voltage withstand capacity of a single series capacitor. This structure of HB-MMC is convenient to expand the capacity of flexible current limiter.

### Mathematical model of HB-MMC

**Fig 2:**
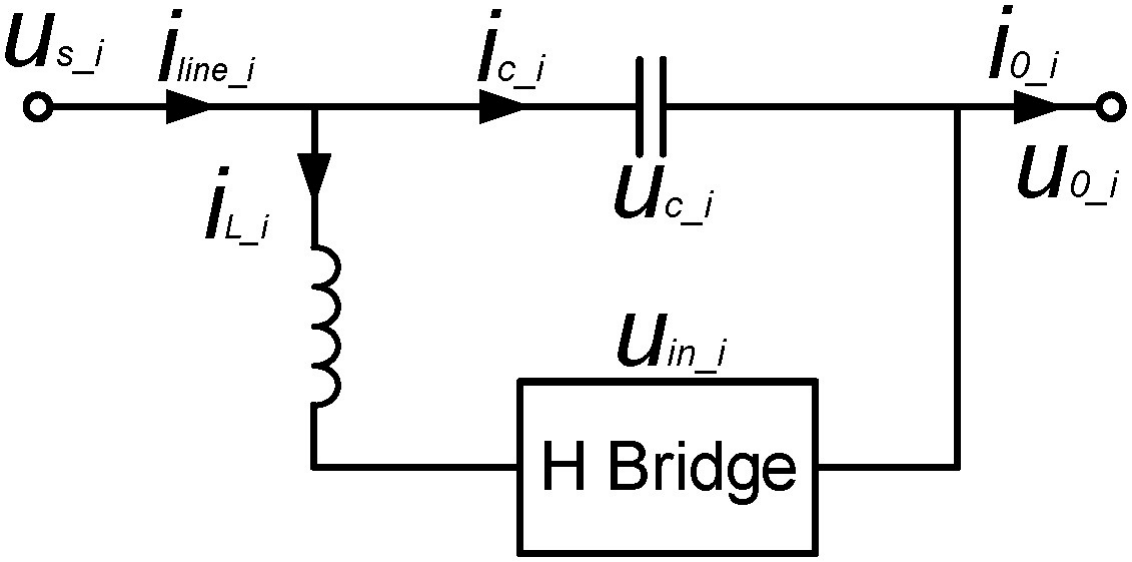
Equivalent circuit model of HB-MMC.

Fig 2 shows the equivalent circuit model of HB-MMC. According to Kirchhoff voltage and current theorems, the state equation of HB-MMC can be expressed as

{i0_i=Ciduc_idt-iL_iu0_i=us_i-(LidiL_idt-uin_i(k))uin_i=∑j=1M(G1-G2)Udc_j (1)

Here, *i*_0_i_ and *i*_line_i_ are the HB-MMC’s output and input current, respectively. *i*_L_i_ and *i*_c_i_ respectively represent the currents of the inductance and capacitance corresponding to the ith HB-MMC. *L*_i_ and *C*_i_ are the inductance and capacitance of the ith HB-MMC, respectively. *u*_c_i_ is the voltage of the ith series capacitance. *u*_in_i_ is the output voltage of the ith HB-MMC and it can be obtained by the unipolar two-valued logic switching function, *G*_k_ (k = 1,2). Each phase bridge arm of the three-phase grid-connected inverter has two switching modes.

When a bridge arm is switched on, *G* = 1, otherwise *G* = 0. Table 1 gives the relationships among these switching modes, *G*_k_ and their output voltages, *u*_in_i_. Then, the voltage of single-phase NFFCL can be obtained as follows

$$u_{NFFCL}=\sum_{i=1}^{N}\left[L_{i}\frac{di_{L\_i}}{dt}-(\sum_{j=1}^{M}G_{k}U_{dc\_j}{)}_{i}\right]$$ (2)

**Table 1:** Relationship between switching states and output voltage component.

Switch	*G*_1_	*G*_2_	*U*_*in_i*_
*S*_1_	0	0	0
*S*_2_	1	0	*U*_dc_
*S*_3_	0	1	-*U*_dc_
*S*_4_	1	1	0

From (2), when the power grid system fails, if the voltage of the NFFCL, u_NFFCL_, is raised to 90% of the system’s rated voltage, it can be considered that the negative impact of the fault on the non-fault area has been basically eliminated. And because of NFFCL’s three-phase independent control structure, either fault case can be handled independently.

### Control principle of CPMC

**Fig 3:**
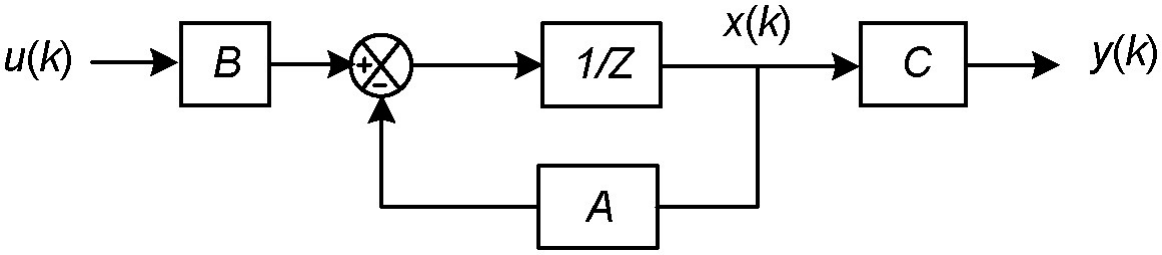
Block diagram of CPMC system.

Fig 3 shows the block diagram of CPMC system [18]. *u*(k) and *y*(k) are the input and output signals in the time of k, respectively. From Fig 3, the transfer functions between *u*(k) and *y*(k), together with the time delay element 1/Z, can be expressed as follows:

y(k+1)=CAx(k)+CBu(k) (3)

Here, *B* and *C* are transfer functions. A is feedback controller. If *u*(k) and *y*(k) are multi-dimensional variables, then

$$\{\begin{array}{l} y_{1}(k+1)=CAx(k)+CBu_{1}(k)\\ \;\vdots \\ y_{i}(k+1)=CAx(k)+CBu_{i}(k)\;(i=1,\cdots ,n)\\ \;\vdots \\ y_{n}(k+1)=CAx(k)+CBu_{n}(k) \end{array}$$ (4)

Therefore, based on (9) and known quantity *u*_*i*_(k), *y*_*i*_(k+1) can be obtained. In order to accurately track the reference value of control system y*_i_(k+1), the value function *f*_i_ is introduced, and it is expressed as

$$f_{i}={\left[y_{i}^{*}(k+1)-y_{i}(k+1)\right]}^{2}$$ (5)

According to (9) and (10), the minimum value function can be obtained

$$f_{min}=min(f_{1},\cdots f_{n})$$ (6)

Then, *y*_*i*_(k+1) corresponding to *f*_min_ will be adopted to the control system, so that the superior performances of control system can be obtained.

### Control strategy of HB-MMC

The single H-bridge converter has four switching modes. The relationships between these switching modes and their output voltages can be obtained from Table 1. If (1) is discretized at the time of (t_k_, t_k+1_), it can be modified as follows:

{iL_i(k+1)=Ts[uc_i(k)-RLLiiL_i(k)+uin_i(k)]Li+iL_i(k)uc_i(k)=us_i(k)-u0_i(k) (7)

Here, T_s_ is one sampling period. L is the inductance, and R_L_ is associated resistance. Based on (1), through logic switching function *G*_1_ and *G*_2_, *u*_*in_i*_ can be modified. Therefore, using each set of switching modes can gain different values of *u*_*in_i*_. If the different values of *u*_in-i_ corresponding to the four switching modes at time k are substituted (7) respectively, four different results of the output current *i*_L*-i*_, of HB-MMC can be obtained.

If HB-MMC’s output current is set as the converter’s control objective, the HB-MMC’s value function *f*_i_ can be expressed as follows [19]:

$$\{\begin{array}{l} f_{i\_1}={\left(i_{L\_i\_ref}-i_{L\_i\_1(k+1)}\right)}^{2}\\ f_{i\_2}={\left(i_{L\_i\_ref}-i_{L\_i\_2(k+1)}\right)}^{2}\\ f_{i\_3}={\left(i_{L\_i\_ref}-i_{L\_i\_3(k+1)}\right)}^{2} \end{array}$$ (8)

**Fig 4:**
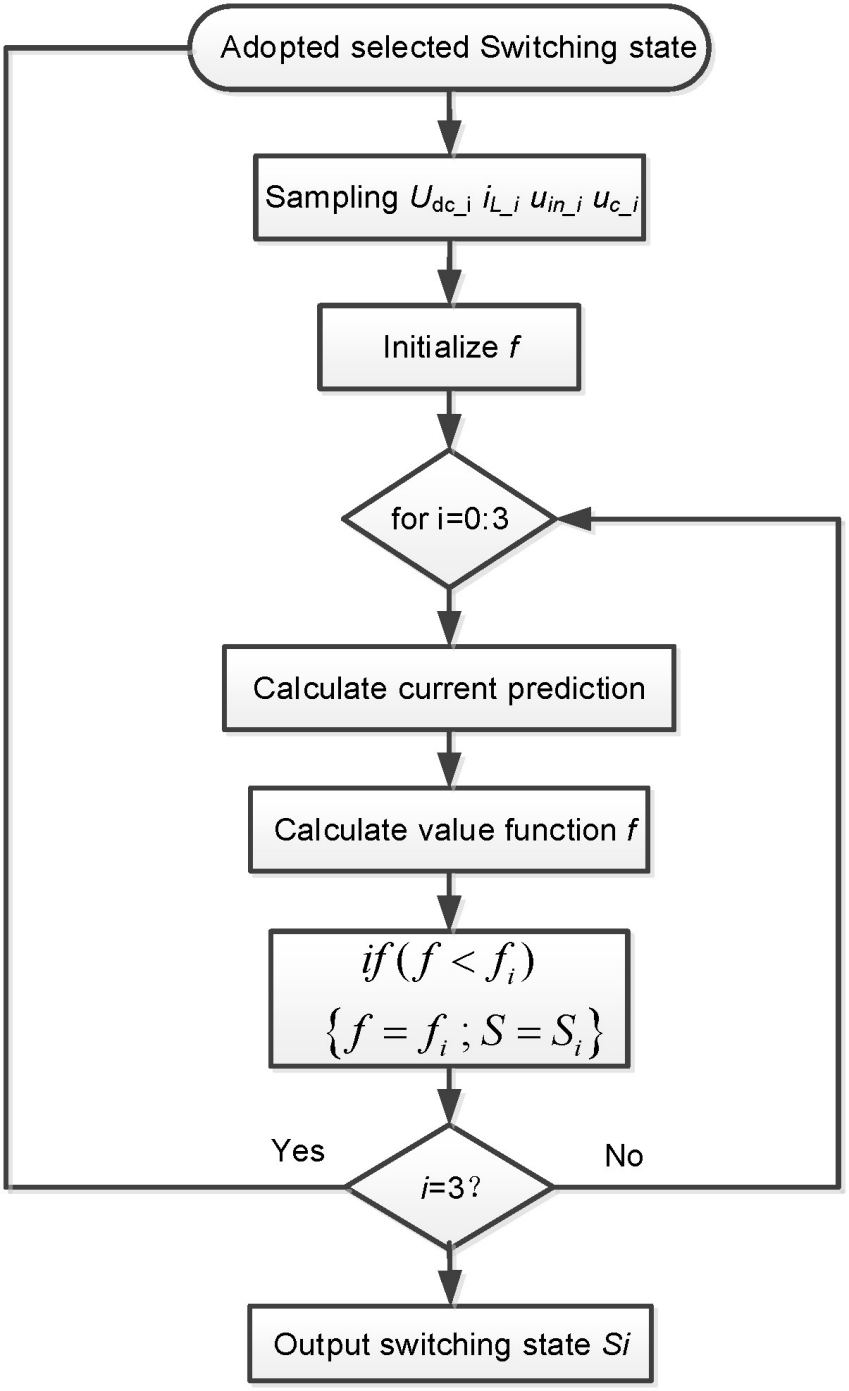
Flow diagram of implemented CPMC system.

Here, *i*_*L-i-ref*_ is the reference current for the ith HB-MMC’s control system. It is worth noting that because the H-bridge converters are connected in series, the value function fi of HB-MMC has a general characteristic, that is, it is suitable for all H-bridge converters of the ith HB-MMC. Therefore, *f*_min_ can be obtained by substituting (8) into (6). Then, *G*_1_ and *G*_2_ corresponding to *f*_min_ will be implemented by H-bridge converters. To further clarify the CPMC algorithm for the H-bridge converter controller, a flow diagram of the CPMC algorithm can be seen from Fig 4 and it is implemented in MATLAB. As can be seen from Fig 4, the voltage signal is sampled at the beginning of the control loop. Then, through (7), the algorithm estimates the current of the HB-MMC and initializes the value of f as a variable that contains the value of the lowest quality function evaluated so far by (8). The strategy then enters a loop, and for the switching state of each possible H-bridge converter, considering the voltage *u*_*in_i*_, the current prediction can be obtained from (1) and (7).

The quality function of (8) is evaluated using the current predicted results. If the evaluated quality function f is stored as *f*_i_, for a given switch state, the lower limit value is stored as *f*_i_, and the switch state number is stored as *S*_i_. After evaluating all four switch states, the loop ends. The state that produces *f*_min_ is identified by the variable *S*_min_ and will be applied to the converter during the next sampling interval, thus starting the CPMC algorithm again.

Therefore, the switching state of the converter can be obtained so that the output current is closest to its reference value.

**Fig 5:**
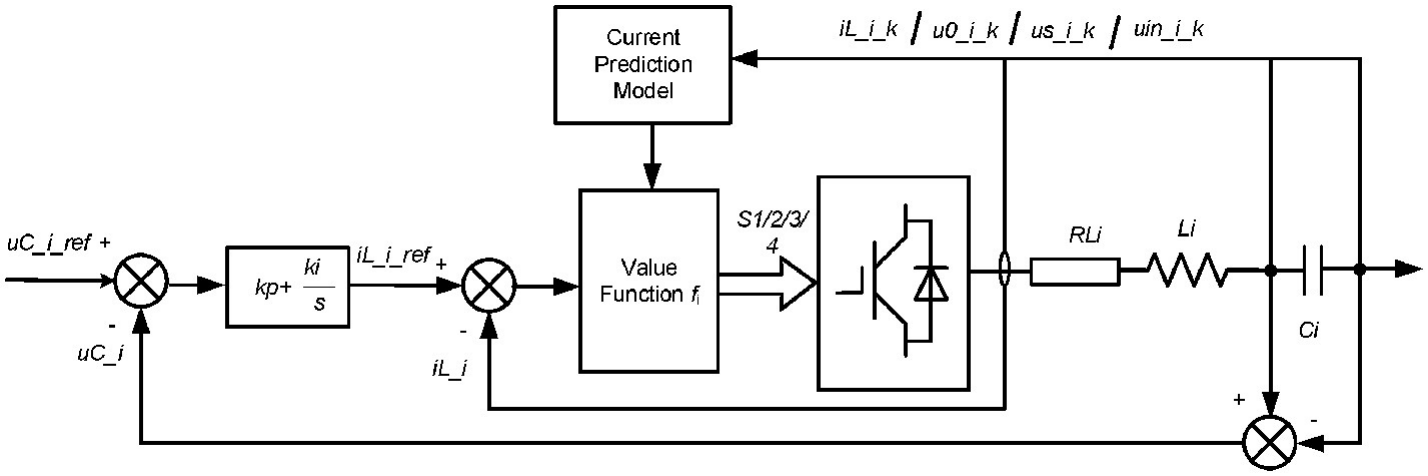
Control strategy of HB-MMC.

Fig 5 shows the control strategy of HB-MMC, together with CPMC system. From Fig 5, under fault conditions, the reference signals of value function, *i*_L_i_ref_, can be expressed as

{iL_i_ref=(uc_i_ref-uc_i)(kp+kis)uc_i_ref=(unM-uL_i)N (9)

Here, *u*_*c-i-ref*_ is the reference voltage of the series capacitance. *u*_*n*_ is the rated voltage of power system. Accordingly, NFFCLs take an active role in voltage fluctuation, so that the electric energy’s quality of multi-source system can be effectively improved.

## Distribution network transient characteristics with NFFCLs

**Fig 6:**
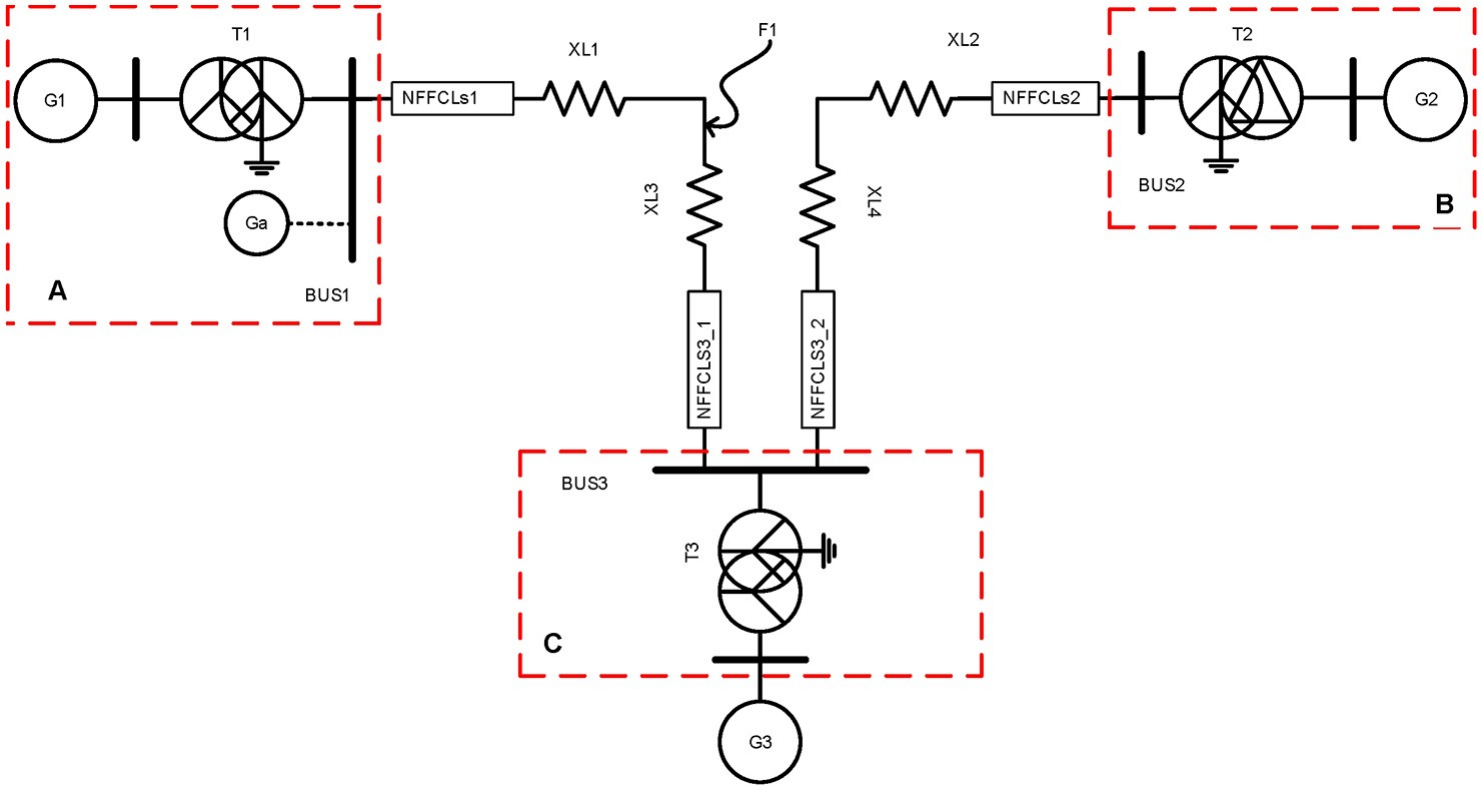
Multi-source distribution network schematic diagram.

Fig 6 shows the schematic diagram of multi-source distribution network. In order to meet the complexity analysis of large power grid, a three-machine system is selected to discuss the current-limiting principle of NFFCL under various fault conditions. Through the approximate method, by calculating the voltage level network of different equipment parameters, it can be used to convert into standard system. Therefore, the impedance’s per-unit value of each equipment can be obtained as [20]:

{XG1=XG2=XG3=X*(N)SBSNXT1=XT2=XT3=UF%100SBSNXL1=x1L1SBUav2;XL2=x2L2SBUav2XL3=x3L3SBUav2;XL4=x4L4SBUav2 (10)

**Fig 7:**
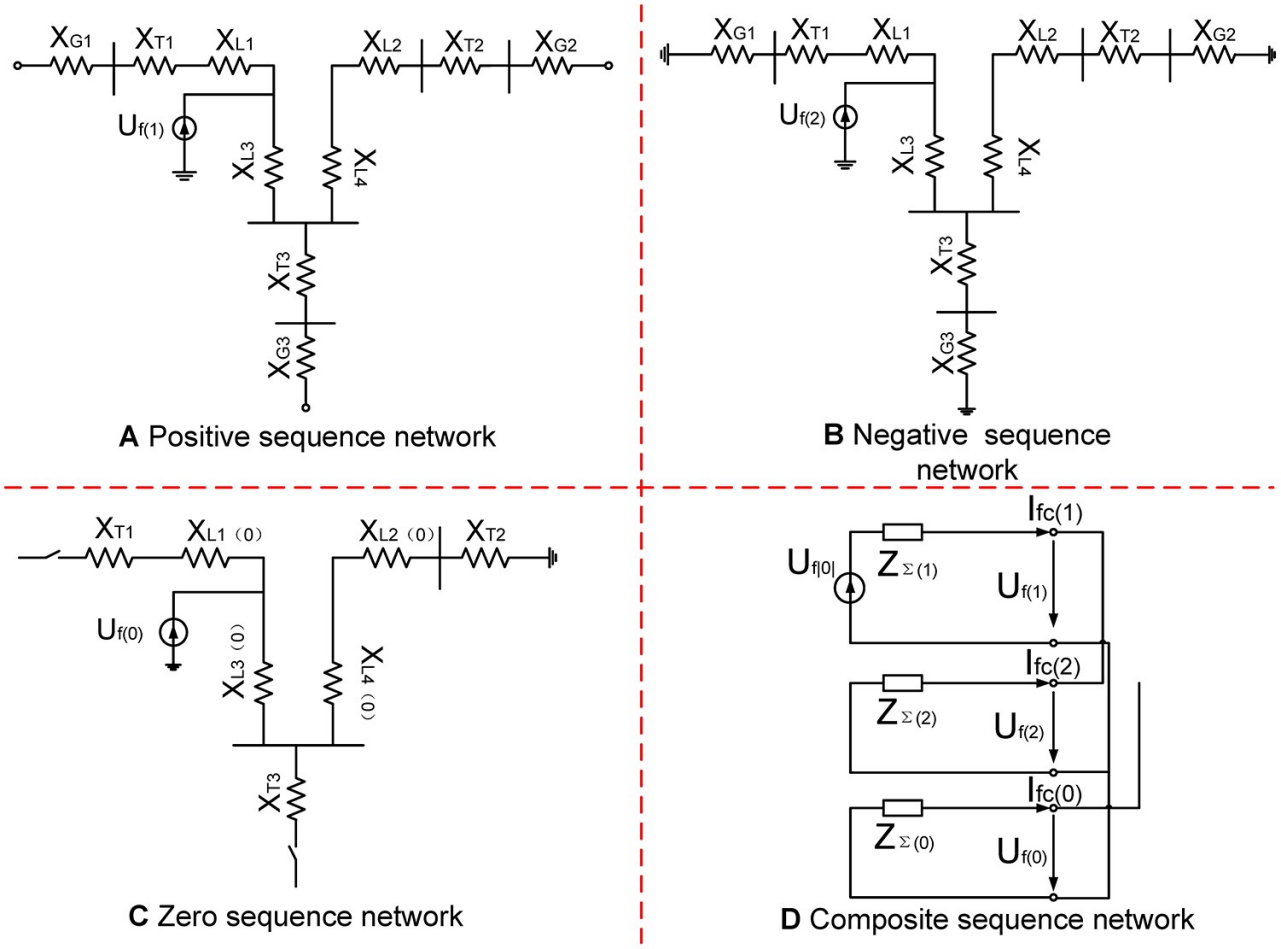
Three-sequence network diagram. (A) Positive sequence network (B) Negative sequence network (C) Zero sequence network (D) Composite sequence work.

Here, ${\overset{*}{X}}_{\left(N\right)}$ is the reactance’s per-unit value. X_GI/G2/G3_, X_TI/T2/T3_ and X_LI/L2/L3_ are the impedance of electric generator (G_1_/G_2_/G_3_), transformer (T_1_/T_2_/T_3_) and transmission line, respectively. S_N_ and S_B_ are the system’s rated power and reference power, respectively. U_F_ is the percentage of short-circuit voltage. x_1_, x_2_, x_3_ and x_4_ are the reactance per unit of length. *L*_1_, *L*_2_, *L*_3_ and *L*_4_ present the length of lines. According to Fig 6, a three-sequence network diagram can be obtained, as shown in Fig 7. Then, the equivalent impedance of three-sequence network diagram can be expressed as:

$$\{\begin{array}{l} Z_{\sum (1)}=\frac{\left(X_{G1}+X_{T1}+X_{L1})*(X_{L3}+\frac{\left(X_{L2}+X_{L4}+X_{G2}+X_{T2})*(X_{G3}+X_{T3}\right)}{X_{G3}+X_{T3}+X_{L2}+X_{L4}+X_{G2}+X_{T2}}\right)}{X_{G1}+X_{T1}+((X_{L1}+X_{L3})+\frac{\left(X_{L2}+X_{L4}+X_{G2}+X_{T2})*(X_{G3}+X_{T3}\right)}{X_{G3}+X_{T3}+X_{L2}+X_{L4}+X_{G2}+X_{T2}})}\\ Z_{\sum (0)}=X_{L2(0)}+X_{L3(0)}+X_{L4(0)}+X_{T2}\\ Z_{\sum (2)}=\frac{\left(X_{G1}+X_{T1}+X_{L1})*(X_{L3}+\frac{\left(X_{L2}+X_{L4}+X_{G2}+X_{T2})*(X_{G3}+X_{T3}\right)}{X_{G3}+X_{T3}+X_{L2}+X_{L4}+X_{G2}+X_{T2}}\right)}{X_{G1}+X_{T1}+((X_{L1}+X_{L3})+\frac{\left(X_{L2}+X_{L4}+X_{G2}+X_{T2})*(X_{G3}+X_{T3}\right)}{X_{G3}+X_{T3}+X_{L2}+X_{L4}+X_{G2}+X_{T2}})} \end{array}$$ (11)

Here, *Z*_∑(1)_, *Z*_∑(2)_ and *Z*_∑(0)_ are the impedance of positive sequence, negative sequence and zero sequence, respectively. When 2LG occurs at F1, fault boundary conditions for 2LG (B phase and C phase) of multi-source distribution networks are described as follows:

{I˙fa=0U˙fb=U˙fc=0 (12)

Here, *I*_fa_ is the fault current of A phase. U_fb_ and U_fc_ are the fault voltage of B and C phases, respectively. Then, according to symmetrical component method, the relationships of three sequence components can be obtained as:

{I˙f(1)+I˙f(2)+I˙f(0)=0U˙f(1)=U˙f(2)=U˙f(0) (13)

Therefore, based on (13) and Fig 7, the voltage of F_1_, ${\dot{U}}_{f|0|}$, can be expressed as:

$${\dot{U}}_{f|0|}=\frac{Z_{\sum \left(1\right)}+\frac{Z_{\sum \left(0\right)}Z_{\sum \left(2\right)}}{Z_{\sum \left(0\right)}+Z_{\sum \left(2\right)}}}{\sqrt{3-\frac{3Z_{\sum \left(0\right)}Z_{\sum \left(2\right)}}{(Z_{\sum \left(0\right)}+Z_{\sum \left(2\right)}{)}^{2}}}}{\dot{I}}_{f}$$ (14)

Here, ${\dot{I}}_{f}$ is fault current. When NFFCLs are connected to a multi-source distribution network, NFFCLs can be equivalent to a voltage source because the output current of its HB-MMC is controlled. And it is assumed that the output current amplitude of HB-MMC is $k{\dot{I}}_{f}$. Then, ${\dot{U}}_{f|0|}$ can be modified as:

$${\dot{U}}_{f|0|}=(\frac{Z_{\sum \left(1\right)}+\frac{Z_{\sum \left(0\right)}Z_{\sum \left(2\right)}}{Z_{\sum \left(0\right)}+Z_{\sum \left(2\right)}}}{\sqrt{3-\frac{3Z_{\sum \left(0\right)}Z_{\sum \left(2\right)}}{(Z_{\sum \left(0\right)}+Z_{\sum \left(2\right)}{)}^{2}}}}+k){\dot{I}}_{f}$$ (15)

Accordingly, together with the above method, the target voltages in other failure conditions can be obtained as:

{U˙f|0|2LS=(Z∑(1)+Z∑(2)3+k)I˙fU˙f|0|3GL=(Z∑(1)+k)I˙fU˙f|0|1GL=(3Z∑(1)+Z∑(2)+Z∑(0)+k)I˙f (16)

Here, ${\dot{U}}_{f}{\left|0\right|}_{2LS},\;{\dot{U}}_{f}{\left|0\right|}_{3GL}$ and ${\dot{U}}_{f}{\left|0\right|}_{1GL}$ present two-phase short faulty, three-phase grounding and single-phase grounding, respectively. From (14) and (15), together with the NFFCLs, the fault voltage ${\dot{U}}_{f}|0|$ can be significantly improved under various fault conditions.

## Simulation verification

To evaluate the performance of the proposed NFFCL, a simulation model is built in MATLAB / SIMULINK based on Figs 1 and 7. And Table 2 offered the simulation parameters. Under normal operating conditions, a current mode harmonic source is connected to the distribution network at BUS1 to verify the harmonic rejection capability of NFFCL. And three different FRT control methods are compared in multi-source distribution networks. As shown in Fig 7, it assumes that 3LG (line to ground) and a 2LG fault occur at F1. The occurrence time and duration of the failure are set to 0.5 s and 400 ms, respectively. Considering the detection delay time of NFFCL, it is assumed that NFFCL is triggered after 10ms. According to international operating standards of distribution networks, this paper gives some judgment indexes whether negative influence is eliminated.

1. When fault occurs there is no obvious voltage distortion (THD < 5%) and fault current is large.
2. Under fault condition, AC bus voltage deviation of non-fault zone is less than 7%U_N_ (U_N_ presents the rated voltage).
3. In addition, under an unbalanced failure condition, voltage unevenness can be controlled within 5%.

**Table 2:** Main simulation parameters of system model.

**Generator**	**Capacity/Voltage**	**X/R**
G1	192MVA/18kV	1e5
G2	128MVA/13.8kV	1e5
G3	247.5MVA/16.5kV	1e5
**Transformer**	**Capacity**	**Ratio**
T1	4.35e6VA	18KV/10.5KV
T2	4.35e6VA	13.8KV/10.5KV
T3	4.35e6VA	16.5KV/10.5KV
**Current Limiter**		
NFFCL		L = 0.2H; C = 2e-4
SFCL_A_		5Ω
SFCL_B_		10Ω

### NFFCLs voltage adjusted ability

**Fig 8:**
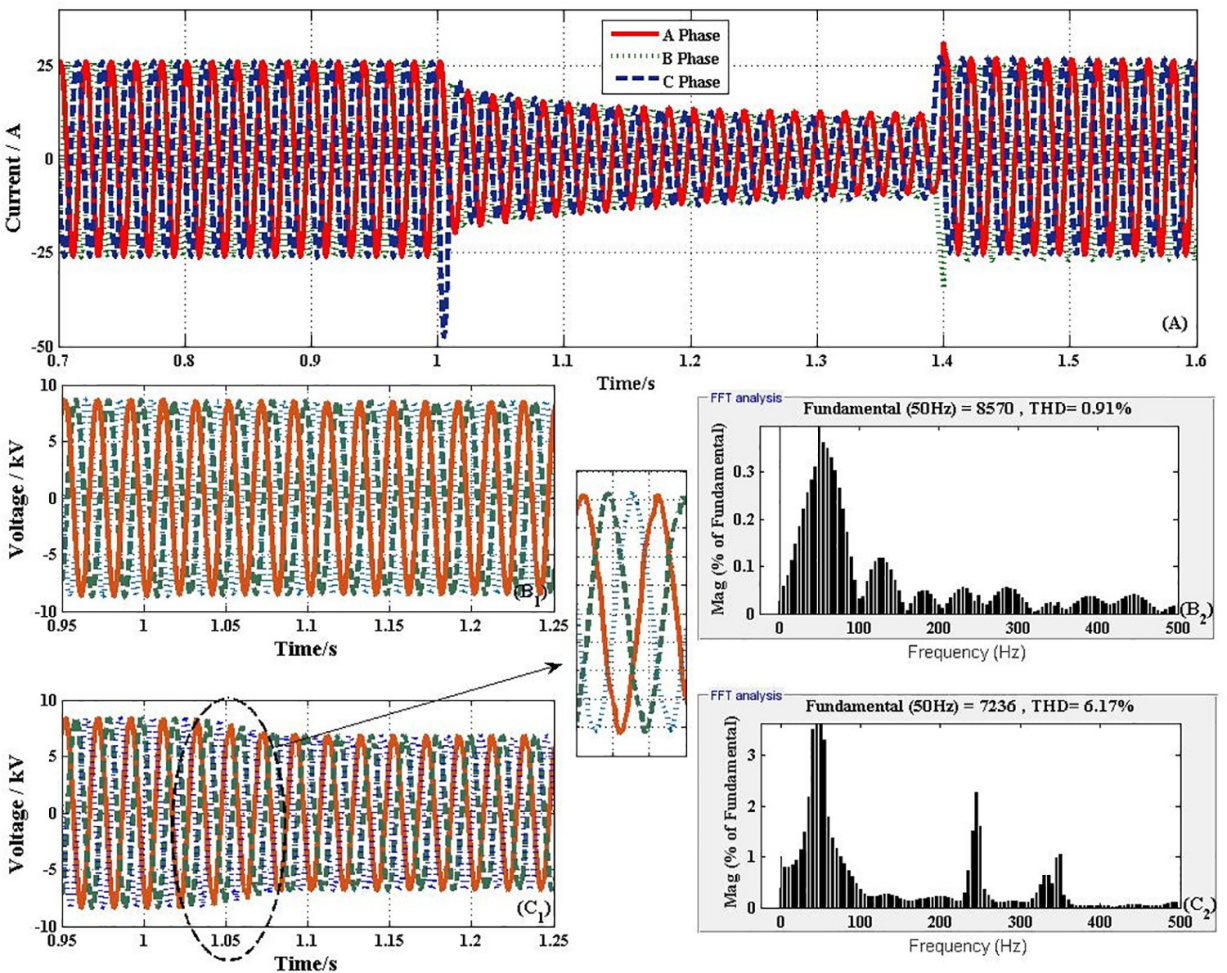
Transient performances curve of the voltage on both sides of NFFCLs_1_. (A) Current through NFFCLs_1_. (B) BUS_3_ harmonic voltage and its THD without NFFCLs. (C) BUS_3_ harmonic voltage and its THD with NFFCLs.

Fig 8 shows the transient performances curve of the voltage on both sides of NFFCLs_1_. Suppose that at 0.5s, the harmonic source G_a_ is connected to the distribution network at BUS_1_, as shown in Fig 6. Because harmonic current (as shown in Fig 8(A)) from G_a_ make voltage’s THD up, NFFCLs play an active power filter to adjusted ability. From Fig 8(B) and 8(C), if the distribution network is with NFFCLs 1, when harmonic sources connect to distribution networks, the line side harmonic voltages of NFFCLs 1 can be significantly suppressed and THD can be reduced from 6.17% to 0.91% (THD < 5%). Therefore, the voltage adjusted function can be realized by NFFCLs_1_.

### 3LG FRT transient performance

**Fig 9:**
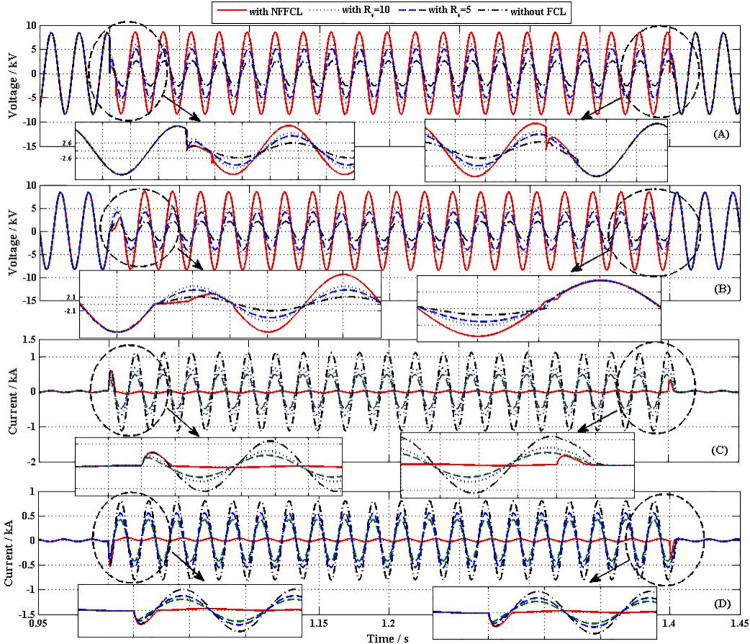
Transient performances curve of distribution network. (A) voltage of BUS1 (B) voltage of BUS3 (C) Output current of BUS1 (D) Output current of BUS3.

In order to verify the correctness and effectiveness of the methods, three different control methods were compared in this paper. These three control methods included (A) with NNFCL, (B) with SFCL, R_SFCL_ = 10Ω, (C) with SFCL, R_SFCL_ = 5Ω and (C) without FCL. Fig 9 shows the transient performances of distribution network, when a 3LG fault happens at F_1_. From Fig 9, without FCL, the voltage of BUS1/BUS3 drop to 2.3kV / 2kV and the fault current is achieved to 1.04kA/0.8 kA. Nevertheless, with the addition of FCL, the voltages of BUS1 and BUS3 are promoted and the fault current is obviously inhibited. From Fig 9(A) and 9(B), when SFCL = 10Ω (or SFCL = 5Ω) is connected into transmission line, the voltages of BUS1 and BUS3 are respectively raised to 6.2 kV and 5.1kV (or 5 kV and 4 kV). And their corresponding fault currents are suppressed to 0.6 kA and 0.55 kA (or 0.45 kA/0.41kA), as Fig 9(C) and 9(D) shown. Therefore, SFCL plays an active role in raising bus voltage and suppressing fault current. But it still can’t completely isolate the failure negative impact on the system.

**Fig 10:**
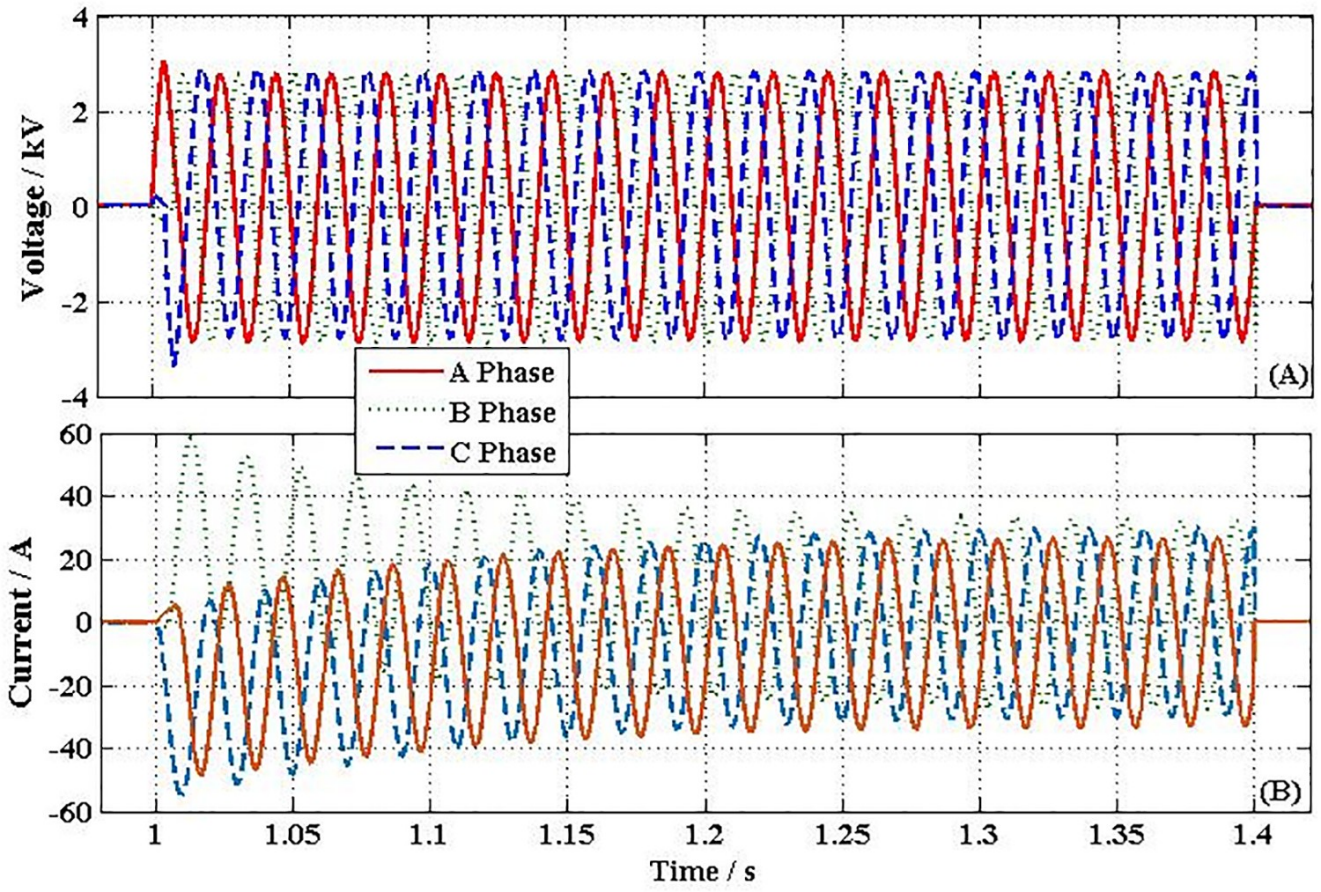
Transient performances curve of NFFCLs_1_. (A) Voltage of single series capacitance (B) Output current of H-bridge circuit.

After triggering NFFCLs_1_ and NFFCLs_3_, the voltages of BUS1 and BUS3 are all raised to rated values (8.16 kV, namely, AC bus voltage deviation significantly less than 7%U_N_) and their corresponding fault currents are restrained to 30A and 25A which are much lower than the currents in the previous two cases. Then, the fault area is isolated by NFFCLs_1_ and NFFCLs_3_, so that the load of non-fault area can run normally. In order to further discuss transient characteristics of NFFCL, the variation curves of H bridge of NFFCLs_1_ are shown in Fig 10(A) and 10(B). From Fig 10(A), the voltage drop of single cascade capacitance is 2.8kV. When the number of the cascade capacitance increased, the voltage drop of single cascade capacitance can be significantly decreased. Therefore, according to the voltage endurance capability of single cascade capacitance, the number of cascade capacitance can be confirmed. From Fig 10(B), it can be seen that maximum current and steady current of H bridge circuit are respectively 52A and 37A, which are within tolerance range.

Therefore, NFFCLs has great potential as a new type of fault isolation equipment. To further clarify performance of proposed strategies in symmetric fault condition, more performance metrics can be obtained from Table 3.

**Table 3:** Comparison of 3LG FRT transient stability.

Current Limiter	U_BUS1_	U_BUS2_	*η*_1_
NFFCL	8.16 kV	8.16 kV	0% / 0%
SFCL_A_	5 kV	4 kV	37.9% / 51%
SFCL_B_	6.2 kV	5.1kV	25% / 37.5%

*η*_1_: voltage sag ratio

### 2LG FRT transient performances

**Fig 11:**
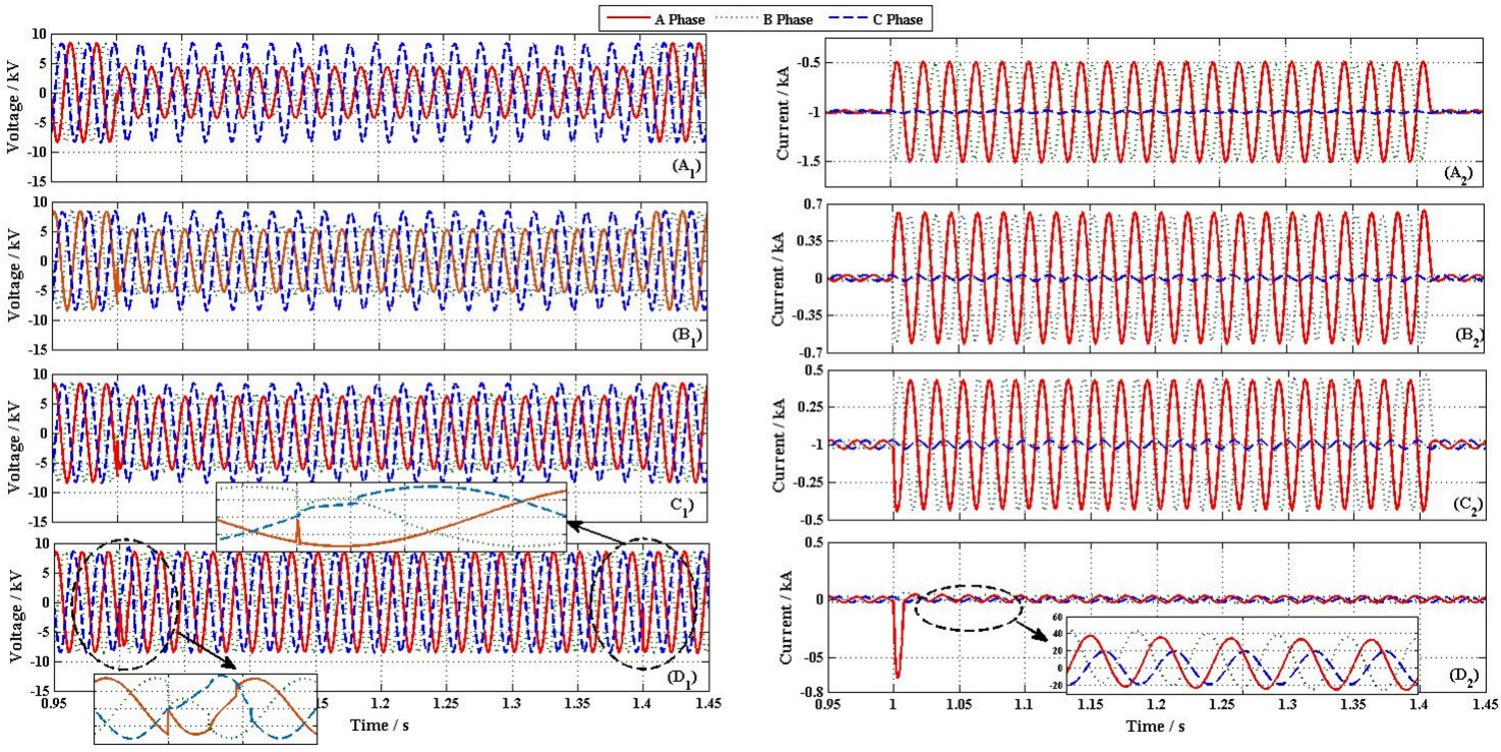
Transient performances curve of distribution network. (A_1_) Voltage of BUS1 without FCL (A_2_) Output current of BUS1 without FCL (B_1_) Voltage of BUS1 with SFCL = 5Ω (B_2_) Output current of BUS1 with SFCL = 5Ω (C_1_) Voltage of BUS1 with SFCL = 10Ω (C_2_) Output current of BUS1 with SFCL = 10Ω (D_1_) Voltage of BUS1 with NFFCLs (D_2_) Output current of BUS1 with NFFCLs.

Fig 11 shows the transient performances of distribution network, when a 2LG fault happened at F_1_. Because BUS1 and BUS3 have similar voltage and current characteristics, this section mainly analyzes output characteristic curves of BUS1 as examples.

From Fig 11, it is found that the unbalanced voltages and currents of BUS1. Similar to the above 3LG, without FCL, the three phase voltages respectively drop to 4.6kV, 3.9kV and 8.16 kV. When SFCLs of A phase and B phase are quenched by fault currents, the A phase and B phase voltage of BUS1 are improved. When the value of SFCL is 5Ω/10Ω, the A phase and B phase voltages of BUS1 respectively are 6kV/6.8 kV and 5.6kV/6.5 kV, as Fig 11(B_1_) and 11(C_1_) shown. Similar to the voltage case, after SFCLs quenching, the fault currents are quickly suppressed from 1.05 kA and 0.98 kA to 0.64 kA/ 0.45 kA and 0.62 kA/0.43 kA, as Fig 11(B_2_) and 11(C_2_) shown.

**Fig 12:**
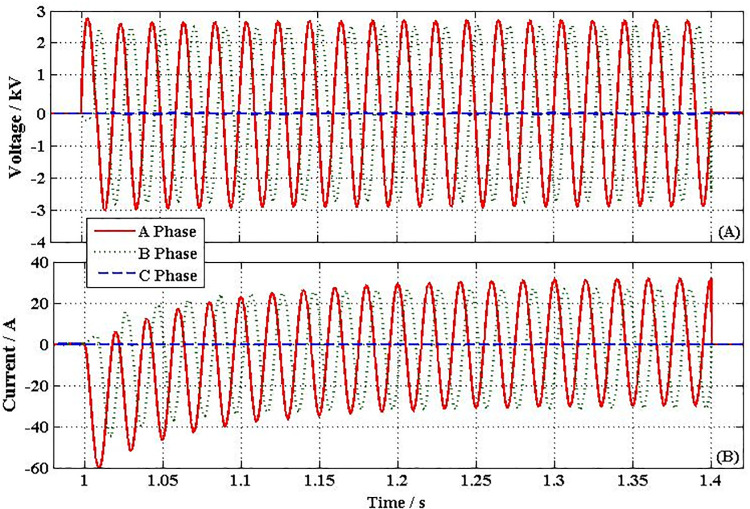
Transient performances curve of NFFCLs_1_. (A) Voltage of single series capacitance (B) Output current of H-bridge circuit.

As shown in Figs 11(D1) and 10C2, after triggering NFFCLs 1 and NFFCLs 3, fault currents are rapidly suppressed and rated voltages on their power side are also available (namely, voltage unevenness less than 5%). In this way, the load in the non-fault area can operate normally. Fig 12(A) and 12(B) show the transient performances curve of NFFCLs1. From Fig 12A, in order to make the three-phase voltage symmetrical, the unbalanced voltage drop (A Phase 2.6 kV/ B Phase 2.51 kV/ C Phase 0 kV) of the cascade capacitor is obtained. Although there are still voltage drop in single cascade capacitor, its drop value can be reduced when the number of cascaded capacitors increases.

From Fig 12B, the three-phase unbalanced current through the H-bridge circuit of the NFFCLs1 can be obtained. In the tolerance range, their maximum current is 60A. Therefore, NFFCL also has better results under asymmetric fault conditions. To further clarify the performance of proposed strategies under asymmetric failures, more performance metrics can be obtained from Table 4.

**Table 4:** Comparison of 2LG FRT transient stability.

Current Limiter	U_BUS1_-A	U_BUS1_-B	U_BUS1_-C	*λ*_max_
Without FCL	4.6kV	3.9kV	8.16 kV	53%
NFFCL	8.16 kV	8.16 kV	8.16 kV	0%
SFCL_A_	6kV	5.6kV	8.16 kV	32%
SFCL_B_	6.8 kV	6.5 kV	8.16 kV	21%

*λ*_max_: Maximum voltage sag ratio

## Conclusions

In order to eliminate the negative influence of distribution network on non-fault area, a new multi-function flexible fault current limiter (MNFFCL) is proposed in this paper. Based on theoretical analysis and simulation verification, the following conclusions are drawn:

1. With CPMC, the inner-loop PI controllers and PWM module are removed.
2. Due to adopting multi capacitor series structure maximum voltage drop on single capacitor can be determined beforehand by quantity of capacitor.
3. NFFCL can completely eliminate fault negative effects on Non-fault zone under symmetric/asymmetric fault conditions. And its filtering function is also effective.
